# Vaccinating Women Previously Exposed to Human Papillomavirus: A Cost-Effectiveness Analysis of the Bivalent Vaccine

**DOI:** 10.1371/journal.pone.0075552

**Published:** 2013-09-26

**Authors:** Hugo C. Turner, Iacopo Baussano, Geoff P. Garnett

**Affiliations:** 1 Department of Infectious Disease Epidemiology, Imperial College London, London, United Kingdom; 2 International Agency for Research on Cancer, Lyon, France; University of Missouri Kansas CIty School of Medicine, United States of America

## Abstract

Recent trials have indicated that women with prior exposure to Human papillomavirus (HPV) subtypes 16/18 receive protection against reinfection from the HPV vaccines. However, many of the original models investigating the cost effectiveness of different vaccination strategies for the protection of cervical cancer assumed, based on the trial results at that time, that these women received no protection. We developed a deterministic, dynamic transmission model that incorporates the vaccine-induced protection of women with prior exposure to HPV. The model was used to estimate the cost effectiveness of progressively extending a vaccination programme using the bivalent vaccine to older age groups both with and without protection of women with prior exposure. We did this under a range of assumptions on the level of natural immunity. Our modelling projections indicate that including the protection of women with prior HPV exposure can have a profound effect on the cost effectiveness of vaccinating adults. The impact of this protection is inversely related to the level of natural immunity. Our results indicate that adult vaccination strategies should potentially be reassessed, and that it is important to include the protection of non-naive women previously infected with HPV in future studies. Furthermore, they also highlight the need for a more thorough investigation of this protection.

## Introduction

Human papillomavirus (HPV) infection is necessary for the development of cervical cancer in women. In the UK, despite a well organised screening programme that achieves high coverage [1], it is estimated that every year 2,890 women are diagnosed with cervical cancer and 1,111 deaths a year are associated with the disease [1]. In addition, HPV has been linked to anal, vulval, vaginal, penile, and oropharyngeal cancers [2]. These cancers would not be detected in the current screening programme. Two high efficacy prophylactic vaccines against HPV have been developed; a bivalent vaccine (CervarixTM) which protects against types 16 and 18, which are responsible for 79% of cervical cancers in the UK [1], and a quadrivalent vaccine (GardasilTM) that also protects against types 6 and 11 (which are associated with anogenital warts).

Since their license, many countries have introduced routine HPV vaccination programmes targeting adolescent girls before sexual debut. Several countries have also implemented catch-up programmes covering older adolescents [3]. For example, in the UK, the Joint Committee on Vaccination and Immunisation recommended a catch-up vaccination programme of girls aged 13 to 17, which started in 2008 [4]. However, though the United States offer HPV vaccination to women up to 26 years of age [5], few countries have offered HPV vaccination to older age groups, and the uptake for the catch-up programmes has been generally low [6,7].

Due to the complexity of HPV transmission and natural history of cervical cancer, as well as the long delay between infection and clinical outcomes, mathematical models are needed to predict the long-term benefits of different vaccination strategies. Many of the older HPV models made the assumption (based on the trial results at that time) that the vaccine only offers protection to women who had not been previously infected (i.e. are naive to infection) [8,9,10,11,12]. However, when the analysis of the phase three bivalent vaccine trial was expanded to include women that were not currently infected but who had previous serological exposure to HPV 16/18 infection (i.e. women who are non-naïve to infection), it was found that the vaccine had a comparably high efficacy in this group of women as the HPV naive women [13]. Similar trial results have also been reported for the quadrivalent vaccine [14]. This is important as the results from models that do not include vaccine protection in non-naive women may underestimate the cost effectiveness of including older age groups in catch-up programmes. This has implications for policy decisions on which age groups should be included in vaccination strategies.

Although many models have since included this protection to non-naive women [15,16,17,18], to the best of our knowledge no other model has been devised to explicitly investigate the benefit of this protection and the implications it has on the cost effectiveness of vaccinating adults using a dynamic transmission model.

We constructed a model that quantifies the potential protection of women with prior exposure to HPV16/18 to investigate the effect of this protection on the cost effectiveness of extending the vaccination catch-up programme to older age groups in the UK. In addition, we explored a range of lower vaccine costs than in previous economic analyses [15], to reflect that the government negotiated vaccine price will likely be lower than the list price assumed in many economic evaluations.

## Materials and Methods

### Model procedures

We developed a deterministic, dynamic transmission model to represent acquisition and heterosexual transmission of infection, with an embedded progression model to represent the subsequent development of HPV-related disease. The model, partly based on Jit et al. [15] was stratified by HPV type, age, gender and sexual activity based risk group. More detail is provided in the File S1.

HPV types in the model were divided into four groups: type 16, type 18, other oncogenic high-risk types with vaccine cross protection and other oncogenic high risk types without vaccine cross protection. We used type specific model compartments to represent women being susceptible to HPV infection, infected with HPV or immune to infection. The susceptible compartments were further subdivided into naive and non-naive to HPV infection. Non-naive women (i.e. women with prior exposure to HPV) were assumed to occupy the susceptible non-naive or naturally immune compartments. The type-specific infected compartments in women were subdivided into being infectious but free of disease, having cervical intraepithelial neoplasias of different grades (CIN I, II, or III), and having invasive squamous cell carcinoma (SCC). We adopted the same structure for adenocarcinomas, with states for cervical glandular intraepithelial neoplasia (CGIN) replacing CIN. A specific precursor lesion state could regress to a less severe state, to the immune state, or to susceptible (non-naive) state, either as a result of natural regression (at rates independent of age) or age-dependent screening and treatment (see Tables S4 and S5 in File S1 for progression/regression and screening rates). Men were assumed only to occupy type-specific model compartments representing HPV susceptible, infected and immune.

The model population consisted of individuals aged between 12 and 74 years old, divided into 10 age classes based on data from the Office of National Statistics [19]. The model population was stratified into three sexual behaviour groups. More detail is provided in Table S2 in File S1.

### Parameter estimation

Using nonlinear least-squares regression the model was fitted to HPV type and age-specific prevalence data [20], by estimating a type-specific transmission probability (See File S1for details). If women were found to be infected with multiple types of HPV in the data, we assumed women to have the most oncogenic HPV type(s) present when fitting the model (i.e. if the data showed a woman was positive to both HPV16 and 18, that person was classified as HPV16 in the model). By estimating the age dependent and HPV type specific progression rate of CIN III to SCC and CGIN III to adenocarcinomas, the model’s predicted cancer incidence was fitted to data on the number of cancer cases reported in the UK and to data describing the distribution of HPV types in cancer cases [21,22]. Double counting of disease outcomes was avoided by attributing cancer to the most oncogenic HPV type(s) present. Due to the lack of knowledge of immunity against HPV, we repeated our model simulations under different assumptions of the level of natural immunity (described in the sensitivity analysis section). The model was refitted for each different natural immunity scenario i.e. for each different immunity assumption we had a different transmission probability and a different cancer progression rate to ensure that the incidence of cancer matched the observed data in all of the scenarios we investigated. The range of parameter values estimated is shown in Table S8 in File S1.

### Vaccination

The model assumed the vaccine gives naive women 100% protection against HPV16/18 infections [23]. In addition, the model assumed that the vaccine has cross protection to the high risk HPV types not included in the vaccine (with a 47% efficacy against infection) based on clinical trial data for the bivalent vaccine [24], and other modelling studies [17]. To quantify the effect of the protection of women non-naive to HPV16/18, we varied their protective vaccine efficacy against infection between 0% and 100%. It was assumed a woman would need at least two doses of the vaccine to receive any protection [25]. For catch-up campaigns, women aged over 16 years were assumed to receive their vaccination through general practice clinics. Dose specific coverage estimates of each age class were matched to reported annual HPV vaccine coverage data (See Table S6 in File S1). All three doses of the vaccine were assumed to be given in the same year [26].

Vaccine-induced protection may wane moving vaccinated individuals to a susceptible (non-naive) compartment. The duration of vaccine protection, which was varied as part of the sensitivity analysis was assumed to be the same for both naive and non-naive women. The vaccine is assumed to offer no benefit to women who are currently infected with HPV providing no effect on an individual’s lesion status and no effect on the rate of HPV clearance [27,28]. The vaccine is assumed to have no effect on non-naive individuals’ risks to other high-risk HPV types.

### Cost effectiveness analysis

We analysed the cost effectiveness of a range of vaccination strategies using a healthcare provider perspective. The baseline scenario was the current UK vaccination programme, i.e. vaccination of girls aged 12-13 using a school based programme and with a catch-up programme of 14-17 year olds as well as screening and treatment of older women (based on the current UK screening programme - see File S1 for details). We investigated a range of other vaccination strategies by varying the age of catch up. We did not explore vaccination programmes targeting adolescent males.

We measured the incremental cost effectiveness ratio (cost per Quality Adjusted Life Year (QALY) gained) of progressively extending the catch-up programme to older age groups, over a 100 year time horizon. This was calculated by dividing the additional cost by the additional benefit of a particular vaccination programme compared to the previous vaccination strategy i.e. the next most expensive option. Our baseline scenario was compared to a programme using screening and treatment only. The unit of effectiveness was QALY gained (details provided in File S1). This analysis used a willingness to pay threshold of £30,000 per QALY gained, which is the standard cut-off value usually used by the National Institute for Health and Clinical Excellence for evaluating health technologies in the UK [29]. See Table 1 for the parameter values for the cost and utility weights. Following the National Institute for Health and Clinical Excellence guidelines, the costs and the benefits were discounted at 3.5% at baseline and the discount rate varied as part of the sensitivity analysis [30].

**Table 1:** Parameters used in economic model.

**Health State**	**Utility**
CIN I	0.91 [31]
CIN II	0.87 [31]
CIN III	0.87[31]
Cancer	0.6* [32]
Cancer Treatment	0.84* [32]
Positive pap smear result received	0.98 [31]
**Screening and Treatment**	**Costs**
Cost per Screening (Pap Smear)	£29.02 [33]
Colposcopy	£173.58[34]
Treatment of precancerous lesions	£383.63[34]
Treatment of cancer	£20231.33* [34,35]
**Vaccine**	**Costs**
Cost per dose	£40 or £20 (estimates)
Administration cost per dose (School based)	£5.30[36]
Administration cost per dose (GP based)	£11.87 [37]

^*^ indicates that the parameters are a weighted average of the four different Federations of Gynecologists & Obstetrician stages.

CIN: cervical intraepithelial neoplasias. CGIN: cervical glandular intraepithelial neoplasia. Prices are adjusted to 2011.

### Sensitivity analysis

In order to investigate the effect of the protection of women non-naive to HPV, we varied their vaccine efficacy between 0% and 100%. To reflect the uncertainty surrounding the natural history of the infection, we performed a multi-way sensitivity analysis on the level of natural immunity; both the proportion that experience immunity (25%, 50%, 75%, and 100%), and the duration of immunity (2 years, 10 years, 20 years, and lifelong) were varied. These simulations were summarised as median values with an interquartile range (IQR) to illustrate the variations in the potential cost-effectiveness of the vaccine depending on the level of natural immunity. Because there is no data providing an estimate of the duration of vaccine induced immunity, we present a set of scenarios with different durations of vaccine- associated immunity (10 years, 20 years, and lifelong). Because the UK government purchased the Cervarix vaccine from GlaxoSmithKline at a negotiated and undisclosed price, we also varied the cost of each vaccine dose (£20 and £40 per dose not including the costs of administering the vaccination, which are described in Table 1and File S1). This assumes that the negotiated price is much lower than the listed price of £80.50 per dose [38]. The discount rate was varied between 0% and 6% [30]. In addition the sensitivity of the results to the assumed vaccination coverage of 12-13 year old school girls was investigated.

## Results

On the basis of our model output’s ‘goodness of fit’ to HPV prevalence data [20], we included 75% of our natural immunity scenarios in our cost effectiveness analysis. The scenarios that we excluded (based on their root-mean-square deviation) notably underestimated HPV16 prevalence, and therefore would have underestimated the vaccine’s impact (see File S1). Table 2 shows the estimated costs that would be incurred and the potential QALYs gained over a 100-year period after the introduction of the vaccine (the results were averaged across the estimates obtained for the different assumptions regarding the level of natural immunity (which is unknown)). The incremental cost of extending the vaccine programme increased with the inclusion of older age groups (see Table 2). However it should be noted that the true incremental cost of extending the vaccine programme will be highly depended on the cost of the vaccine. The vaccination programme generated some cost savings to the health service (approximately £336 million for the current UK strategy) by reducing the number of treatments (for precancerous lesions and cervical cancers), but these savings were outweighed by the cost of the vaccination programme itself.

**Table 2:** Discounted additional costs (£ **millions**)** and quality adjusted life years (QALYs) gained by progressively extending the vaccination catch-up programme against HPV** (**over a 100 year time horizon, and using a** 3.5% **discount rate for costs and benefits**).

Incremental QALYs gained:				
**Vaccination Programme:**	**Mean incremental QALYs gained (total)**	**Mean incremental QALYs gained due to cancers prevented**	**Mean incremental QALYs gained due to reduced treatment**	**Median incremental QALYs**
Ages 12-17	88,392*	52,313*	36,079*	90,108 (77,904-95,175)*
Ages 12-19	4,103†	2,428†	1,675†	4,424 (3,980-8765) †
Ages 12-24	7,979†	4,722†	3,257†	8,318 (7,750-10,049) †
Ages 12-29	4,927†	2,916†	2,011†	5532 (5,169-6,623) †
Ages 12-34	3,334†	1,973†	1,361†	3,347 (3,101-4,136) †
**Incremental cost:**				
**Vaccination Programme:**	**Mean incremental cost of programme**	**Mean incremental net cost**	**Mean incremental cost saved**	**Median incremental net cost**
Ages 12-17	£884*	£538*	£336*	£523 (514-565) *
Ages 12-19	£76†	£57†	£19†	£55 (54-57) †
Ages 12-24	£180†	£145†	£35†	£144 (142-148) †
Ages 12-29	£176†	£162†	£14†	£160 (158-163) †
Ages 12-34	£174†	£172†	£2†	£172 (168-174) †

The vaccine was assumed to cost £20 per dose, last an average of 20 years and provide protection to women with previous exposure to HPV (100% efficacy). The median and mean results are averaged across the estimates for different assumptions of natural immunity (the 1^st^ and 3^rd^ quartiles are shown in brackets). All programmes assume routine vaccine of 12-13 year old girls. *QALY: Quality adjusted life year.*

^*^ Costs or benefits compared to a programme only using screening and treatment

^†^ Costs or benefits compared to the previous vaccination option i.e the next most expensive option

The effect of including the protection of non-naive women on the cost effectiveness of vaccinating 12-13 years olds was negligible. However, it substantially increased the cost effectiveness of vaccinating older women in catch-up programmes. The outcome of the cost effectiveness analysis was also highly dependent on the price of the vaccine and the average duration of protection provided by the vaccine. The level of natural immunity was also found to be inversely related to the cost effectiveness of vaccination, with the higher the level of natural immunity the lower the cost effectiveness of vaccination. The variation in the incremental cost effectiveness ratios caused by the different natural immunity assumptions increased with the age of the group being vaccinated (the median results averaged across the estimates for different assumptions of natural immunity are shown in Table 3). In addition, when higher levels of natural immunity are assumed, the protection of non-naive women has a lower beneficial impact on the cost effectiveness of the vaccine.

**Table 3:** The median incremental cost effectiveness ratios of alternative vaccination catch-up programmes.

**£20 per dose**		
	**Protection to non-naive women**	**Absence of protection to non-naive women**
**Vaccination Programme:**	**Median** (**IQR**)	**Median** (**IQR**)
***20 years’ vaccine protection:***		
Ages 12-17 (Current)	£4,089 (3,981-5,910)*	£4,101(4,027-5,774)*
Ages 12-19	**£14,691 (12,820-20,047**) †	**£20,380 (17,999-29,587**) †
Ages 12-24	**£22,286 (19,093-36,929**) †	£39,849 (35,691-55,984) †
Ages 12-29	£51,816 (41,723-74,516) †	£116,327 (106,273-147,262) †
Ages 12-34	£103,156 (77,921-159,226) †	£335,481 (311,499-406,802) †
***Lifetime vaccine protection***:		
Ages 12-17 (Current)	£1,627 (1,525-1,922)*	£1,801 (1,714-2,001)*
Ages 12-19	**£10,433 (9,110-12,455**) †	**£16,769 (14,533-20,947**) †
Ages 12-24	**£16,557 (14,126-17,852**) †	£34,839 (30,060-42,360) †
Ages 12-29	£33,897 (30,850-36,915) †	£105,637 (90,244-118,537) †
Ages 12-34	£50,125 (39,723-58,561) †	£254,191 (200,629-324,487) †
**£40 per dose**	**Protection to non-naive women**	**Absence of protection to non-naive women**
**Vaccination Programme:**	**Median** (**IQR**)	**Median** (**IQR**)
***20 years’ vaccine protection:***		
Ages 12-17 (Current)	£9,476 (8,145-13,357)*	£9689 (8315-13521)*
Ages 12-19	**£22,268 (17,152-33,507**) †	£38210 (33854-52774) †
Ages 12-24	£36,578 (31,321-60,042) †	£70523 (63236-96679) †
Ages 12-29	£90,320 (73,986-128,491) †	£197865 (180770-249516) †
Ages 12-34	£162,040 (124,938-224,179) †	£563025 (522743-682016) †
***Lifetime vaccine protection***:		
Ages 12-17 (Current)	£3,675 (3,317-4,694)*	£3,802 (3,452-4,856)*
Ages 12-19	**£21,623 (18,404-25,006**) †	**£32,078 (27,300-38,695**) †
Ages 12-24	**£29,021 (27,203-33,887**) †	£62,011 (53,133-74,739) †
Ages 12-29	£60,394 (55,569-66,138) †	£179,880 (153,387-201,579) †
Ages 12-34	£80,278 (65,898-105,678) †	£553,911 (473,101-531,305) †

The incremental cost effectiveness ratios in the presence and absence of protection to non-naive women are shown. No strategies were dominated or extendedly dominated. The median results averaged across the estimates for different assumptions of natural immunity are presented (the 1^st^ and 3^rd^ quartiles are shown in brackets). The costs and benefits have been discounted at 3.5% a year. All programmes assume routine vaccine of 12 year old girls. Median results under the £ 30,000 threshold are shown in bold.

IQR: Interquartile range.

^*^ Cost effectiveness of particular option compared to a programme only using screening and treatment.

^†^ Ratio of additional costs and benefits of particular vaccination programme compared with previous option i.e the next most expensive option.

**Figure 1:**
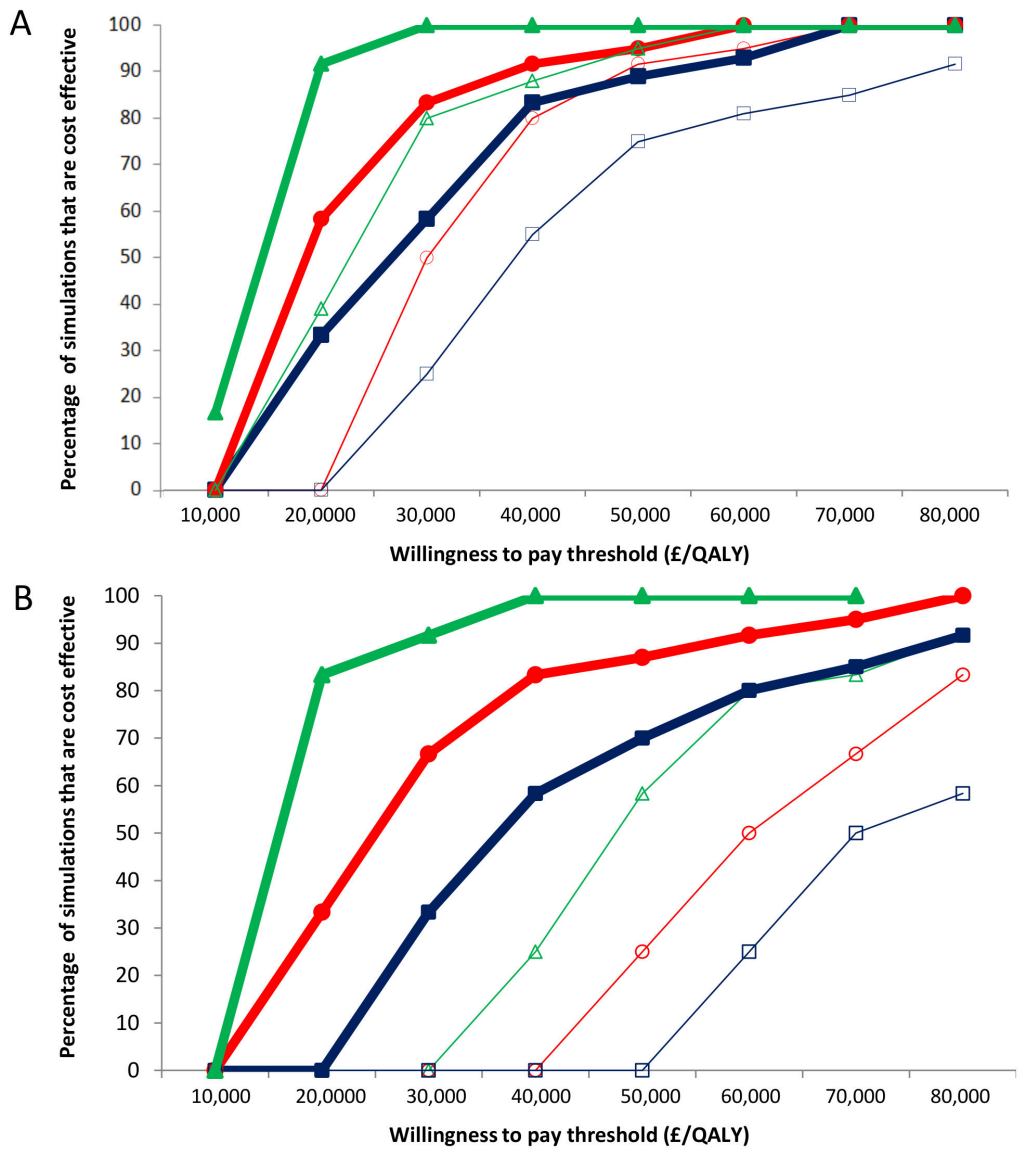
Cost Effectiveness Acceptability Curves for Extending the Vaccination Catch-up to (A) 19 year olds and (B) 24 year olds. Different durations of vaccine induced immunity; Life (Δ), 20 years (○), 10 years (□). Thick lines represent presence of protection to HPV non-naive women and thin the absence. The results presented assumed the vaccine cost is £20 per dose (not including the cost of administering the vaccine) a 100 year time horizon and 3.5% discount rate for costs and benefits. QALY: Quality adjusted life year.

When assuming the presence of protection to non-naive women, the majority of simulations for extending the vaccine programme to include 18 and 19 year olds were cost effective (using a threshold of £30,000 per QALY gained). This extension strategy was not found to be cost effective in the absence of protection to non-naive women, if the cost per dose was £40. The cost effectiveness acceptability curves for extending the catch-up programme to 19 year olds are shown in Figure 1A.

Furthermore, when assuming the vaccine provides 20 years of protection and a cost per dose of £20, extending the catch-up programme further to include 20-24 year olds was found to be cost effective (£22,286 per QALY gained) in the presence of protection to non-naive women. However, this was not cost effective in the absence of the protection to non-naive women (£39,849 per QALY gained). In addition, when the vaccine was assumed to provide both lifelong protection and protection to non-naive women, the results for this strategy were highly cost effective if the vaccine costs £20 per dose (£16,557 per QALY gained), and borderline cost effective if the vaccine costs £40 per dose (£29,021 per QALY gained). However, in the absence of the protection to non-naive women the cost effectiveness decreased substantially (£34,839 and £62,011 per QALY gained respectively). The cost effectiveness acceptability curves for extending the catch-up programme to 25 year olds are shown in Figure 1B. Strategies targeting women over 25 were only found to be borderline cost effective with a vaccine cost of £20 per dose and assuming the vaccine provided both lifelong protection and full protection to non-naive women.

When assuming a lower coverage of the school based programme (targeting 12-13 year olds), the cost effectiveness of extending the vaccine programme to include older women notable increased (see Figure S4 in File S1).

## Discussion

Our economic analysis indicates that the effect of including the protection of women non-naive to HPV on the cost effectiveness of vaccination of 12-13 years is negligible (likely due to the low number of women that have experienced infection in this age group). However, this protective effect can have a substantial effect on the outcome of the cost effectiveness of vaccinating older women in catch up programmes. This was particularly evident in 18-25 year olds, who are not often included in vaccination programmes in Europe [3]. It is worth noting that the impact of including vaccine protection to non-naive women on the vaccine’s cost-effectiveness ratios is affected by the level of natural immunity in the population (with lower benefits if a higher level of natural immunity is assumed). This should be considered in future modelling studies incorporating this protection. When assuming the vaccine provides protection to non- naive women, we assumed that efficacy against infection was the same as that for naive women (i.e. 100%), which may be an overestimate [13]. We therefore varied the efficacy in non-naive women as part of our sensitivity analyses and found that even with a lower value of 50% efficacy, the protection of non-naive women still had a substantial benefit on the cost effectiveness of vaccinating adults (see Figures S5 and S6 in File S1). However, it should be noted that further investigation of this protection and how it may differ from that experienced by HPV naive women is essential.

Our estimates of the cost effectiveness ratios of vaccinating over 18 year olds were substantially lower than those found by the model which informed the UK vaccination strategy, which also took into account this protection to non-naive women [15]. This may be due to the fact that we assumed a lower (estimated) government negotiated price for the vaccine (rather than the vaccine list price of £80.50 per dose) [38]. A study by Bogaards et al. (2011) found that including vaccine protection of non-naive women did not have much bearing on the cost-effectiveness analysis [18]. However, a possible reason for this difference is that their assumptions of vaccine efficacy were only tested as part of a univariate sensitivity analysis, and therefore the effect the level natural immunity has on the projected additional benefits of including the protection of non-naive women were not accounted for [18]. In addition, the study did not account for the effect the increased vaccine protection of non-naive women would have on herd immunity [18].

One of the key strengths of this analysis is that we performed a variety of different simulations, both with and without protection of non-naive women, allowing the additional benefit to be quantified. In addition, dose specific coverage estimates of each age class were matched to reported UK annual HPV vaccine coverage data, capturing the increased dropout rate of the age groups included in the current catch-up programme [6]. However, it should be noted that it is possible that the dropout rate might increase for women aged >18, which requires further investigation. In addition there were some limitations to our approach. We modelled the progression and transmission of each HPV type using separate models (using the method described by [39]). When using separate models, the progression of multiple typed lesions are attributed to the most oncogenic HPV types and this can potentially cause some lesions to be misattributed to the wrong HPV type. However it is not currently possible to accurately parameterize a model for each of the high risk HPV types not included in the vaccine. Although further investigation and quantification of the vaccines impact on other HPV related cancers is important, due to the uncertainties inherent to the progression from HPV infections to these cancers and the lack of available data, we did not incorporate them into our model (therefore the overall health impact from vaccination may be underestimated). Additionally, the model assumed that CGIN lesions were not detected by screening, but in practice some can be detected [40]. Furthermore we did not investigate possible strategies involving vaccinating male adolescents, which though are not used in the UK, are currently being used in Austria and the USA [41]. Based on data reported by Kreimer et al. [25], it was assumed that two doses of the vaccine provide full protection. However it is currently unknown how the number of doses received relates to the duration of vaccine derived immunity (and how much protection is gained by only receiving one dose). More data to inform these parameters in models will be essential for more accurate estimates of the cost effectiveness of different control strategies. Unfortunately the exact price of the vaccine in the UK has not been disclosed publicly. We assume the vaccine price that the government negotiated is lower than the list price of £80.50 per dose [38]. However it is possible that the negotiated price is higher that estimated. It should be noted that an alternative possibility, not accounted for in the model, is that a specific biological factor modulates the capacity of some women to repeatedly clear an infection, which would affect the added benefit of the protection of non-naive women. Furthermore we assumed that the duration of vaccine and natural derived immunity were independent of each other, though it is possible that vaccination may boost the duration of natural immunity. However, there is currently insufficient data to accurately parameterize such features in an HPV transmission model.

The results of this study indicate that the protection of women non-naive to HPV provided by the vaccine has a substantial effect on the cost effectiveness of HPV vaccination catch-up programmes. This was particularly evident in 18-25 year olds for which the results indicate that if the negotiated vaccine cost is below £40 per dose it may be cost effective to extend the UK’s catch-up programme (when assuming comparably high vaccine efficacy in HPV non-naive and naive women). This suggests that if the price of the vaccine is less than £40 per dose, the Department of Health in the UK should reconsider either extending the current catch-up programme or providing a subsidy reducing the cost of private vaccination for women aged 18 to 25. In addition we found that the cost effectiveness of extending the vaccine programme to include older women notably increased when assuming a lower achieved coverage of 12-13 year olds in the school based programmes. This highlights the potential value of this strategy in areas which are currently only attaining a low coverage. However, it should be noted that it is plausible that extending catch-up vaccination to older age groups may lead to women delaying vaccination and this would require further investigation. In addition it is important to consider that offering vaccination to older age groups could potentially lead to a stigmatisation of the school based vaccination programmes (people might believe that only girls that are "planning on their sexual debut" would have the vaccine, potentially decreasing the achieved coverage). In late 2012, the UK’s Department of Health switched to the quadrivalent vaccine, which also includes protection against HPV 6/11 (linked to anogenital warts) [42]. A detailed modelling comparison of the cost effectiveness of the two vaccines is presented in Jit et al. (2011) [17]. However, even though our study was based on the bivalent vaccine and on the UK screening programme (so our cost effectiveness estimates may not be directly generalizable to other counties with different screening and vaccine programmes) it still highlights the importance of both how this protection of non-naive women (which has been found for both vaccines) and how lower government negotiated vaccine prices, may affect the cost effectiveness of vaccinating adult women.

Our modelling projections indicate that the protection of non-naive women can have a profound effect on the outcomes of a cost effectiveness analysis of vaccinating adults. This indicates that adult vaccination strategies should potentially be reassessed and demonstrates the importance of including the protection of non-naive individuals in future studies investigating different HPV vaccination strategies such as male vaccination. In addition, this highlights the need for a more thorough investigation of this protection.

## Supporting Information

File S1 **Model Description and Supplementary Results.** Table S1, Parameter definitions. Table S2, Number of new partnerships per year stratified by age and risk group. Table S3, Population size and death rates stratified by age group. Table S4, Screening and treatment rates stratified by age group and neoplastic status. Table S5, Lesion specific progression and regression parameters. Table S6, Vaccination Coverage for each dose stratified by age. Table S7, Effect of altering discount rate on median incremental cost effectiveness ratio. Table S8, HPV type specific incidence and transmission probability. Figure S1, A Flow Diagram of the Model. Figure S2, The differential equations of the model. Figure S3, Model estimates of age specific prevalence of HPV-16 compared to trial data to which it was fitted. Figure S4, Cost effectiveness acceptability curves for extending the vaccination catch-up programme up to 24 year olds (assuming a lower coverage of the school based programme targeting 12-13 year olds). Figure S5, Cost effectiveness acceptability curves for extending the vaccination catch-up programme up to 24 year olds (assuming 75% vaccine efficacy for non-naive women). Figure S6, Cost effectiveness acceptability curves for extending the vaccination catch-up programme up to 24 year olds (assuming 50% vaccine efficacy for non-naive women).
(DOC)
