# Supplementary material for: Vaccinating Women Previously Exposed to Human Papillomavirus: A Cost-Effectiveness Analysis of the Bivalent Vaccine
Source: PLoS One. 2013 Sep 26;8(9):e75552. doi: 10.1371/journal.pone.0075552 (PMC3784449; doi:10.1371/journal.pone.0075552)
Supplement: File S1 — Model Description and Supplementary Results. Table S1, Parameter definitions. Table S2, Number of new partnerships per year stratified by age and risk group. Table S3, Population size and death rates stratified by age group. Table S4, Screening and treatment rates stratified by age group and neoplastic status. Table S5, Lesion specific progression and regression parameters. Table S6, Vaccination Coverage for each dose stratified by age. Table S7, Effect of altering discount rate on median incremental cost effectiveness ratio. Table S8, HPV type specific incidence and transmission probability. Figure S1, A Flow Diagram of the Model. Figure S2, The differential equations of the model. Figure S3, Model estimates of age specific prevalence of HPV-16 compared to trial data to which it was fitted. Figure S4, Cost effectiveness acceptability curves for extending the vaccination catch-up programme up to 24 year olds (assuming a lower coverage of the school based programme targeting 12-13 year olds). Figure S5, Cost effectiveness acceptability curves for extending the vaccination catch-up programme up to 24 year olds (assuming 75% vaccine efficacy for non-naive women). Figure S6, Cost effectiveness acceptability curves for extending the vaccination catch-up programme up to 24 year olds (assuming 50% vaccine efficacy for non-naive women). (DOC) [file pone.0075552.s001.doc]

**Su**pplementary material

| Figure S1: A Flow Diagram of the Model. | **Page 1** |
| --- | --- |
| Table S1: Parameter definitions | **Page 2** |
| Figure S2: The differential equations of the model | **Page 3** |
| Model description | **Page 4** |
| Sexual behaviour | **Page 4** |
| [Table S2: Number of new partnerships per year stratified by age and risk group](#RANGE!_ENREF_1) | **Page 5** |
| Table S3: Population size and death rates stratified by age group | **Page 5** |
| Screening and treatment description | **Page 6** |
| Table S4: Screening and treatment rates stratified by age group and neoplastic status | **Page 6** |
| Table S5: Lesion specific progression and regression parameters | **Page 7** |
| Quality adjusted life years (QALYs) | **Page 7** |
| Figure S3: Model estimates of age specific prevalence of HPV-16 compared to trial data to which it was fitted | **Page 8** |
| Table S6: Vaccination Coverage for each dose stratified by age | **Page 9** |
| Vaccine Costs | **Page 9** |
| Table S7: Effect of altering discount rate on median incremental cost effectiveness ratio | **Page 9** |
| Parameter estimation | **Page 10** |
| Table S8: HPV type specific incidence and transmission probability | **Page 10** |
| Figure S4: Cost effectiveness acceptability curves for extending the vaccination catch-up programme up to 24 year olds (assuming a lower coverage of the school based programme targeting 12-13 year olds). | **Page 11** |
| Figure S5: Cost effectiveness acceptability curves for extending the vaccination catch-up programme up to 24 year olds (assuming 75% vaccine efficacy for non-naive women). | **Page 12** |
| Figure S6: Cost effectiveness acceptability curves for extending the vaccination catch-up programme up to 24 year olds (assuming 50% vaccine efficacy for non-naive women). | **Page 13** |
| References | **Page 14** |

*
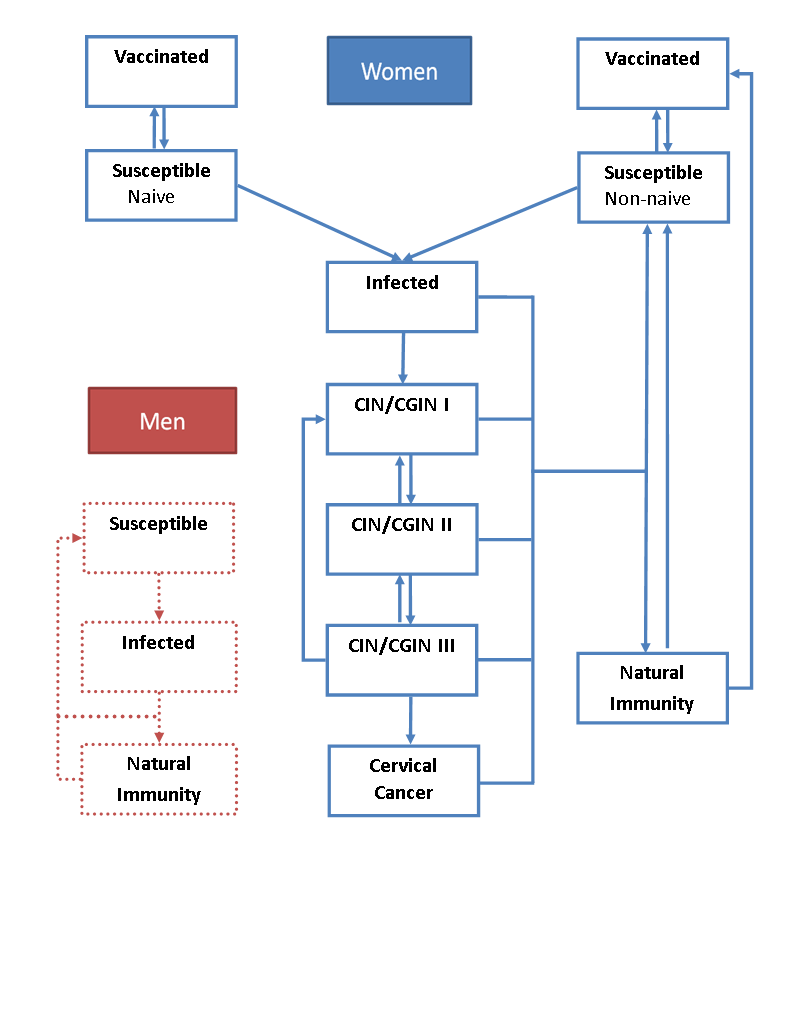
*

**Figure S1: A Flow Diagram of the Model.** *A: Women (solid lines), B: Men (dashed lines)***.** *CIN: cervical intraepithelial neoplasias. CGIN: cervical glandular intraepithelial neoplasia. Cervical cancer includes both invasive squamous cell carcinomas and adenocarcinomas. The model structure was repeated for each of the HPV subtypes modelled. When calculating the cost of vaccination, all women were multiplied by the age and dose specific coverage estimates (not just the susceptible/those with natural immunity).*

### Table S1: Parameter definitions

| Population |
| --- |
| μ: Rate of entry into sexually active population |
| θ:Death rate |
| Nm: Total number of men |
| Nw: Total number of women |
| λ: Force of infection |
| $\Xi$: Excess mortality of cervical cancer |
| Rate of progression to CIN/CGIN and Cancer |
| α: Infected to CIN/CGIN I |
| δ: CIN/CGIN I to CIN/CGIN II |
| γ: CIN/CGIN II to CIN/CGIN III |
| ζ: CIN/CGIN III to invasive cancer |
| Rate of clearance of infection and CIN/CGIN |
| ρ1: Infected to recovered |
| ρ2:CIN/CGIN I to recovered |
| ρ3: CIN/CGIN II to recovered |
| ρ4: CIN/CGIN III to recovered |
| Rates of regression |
| δr: CIN/CGIN II to CIN/CGIN I |
| γr: CIN/CGIN III to CIN/CGIN II |
| γr2: CIN/CGIN III to CIN//CGIN I |
| Natural Immunity |
| π: Rate of waning immunity |
| x:Proportion that develop immunity |
| Treatment |
| tr1: Treatment rate of CIN I |
| tr2: Treatment rate of CIN II |
| tr3 : Treatment rate of CIN III |
| tr4: Treatment rate of Cancer |
| Vaccination |
| Coverage: Proportion of population vaccinated |
| π2: Rate of waning vaccination protection |
| vr: Vaccination rate |
| Abbreviations: CIN, cervical intraepithelial neoplasias; CGIN, cervical glandular intraepithelial neoplasia |

***Figure S2: The Differential Equations of the Model.*** *V^1^; Vaccinated Naive women,* *V^2^; Vaccinated Non-Naive women, S^1^; Susceptible Naive women , S^2^; Susceptible Non-Naive women, I; Infected,**C^1^;CIN/CGIN I,* *C^2^;CIN/CGIN II,**C^3^; CIN/CGIN III,* *C^4^;* *Cervical cancer,**R; Immune, S^m^; Susceptible (men),* *I^m^; Infected (men)**R^m^; Immune (men). Subscripts - Age groups; i, Sexual activity groups; l, Gender; k, lesion and cancer type.* *The differential equations were repeated for each of the HPV subtypes*. *The parameter definitions are shown in Table S1.*

**Model description**

We used separate HPV type-specific model compartments to represent women being susceptible to HPV infection, infected, vaccinated, naturally immune, having precursor lesions (CIN/CGIN I-III), and having cervical cancer (further subdivided into adenocarcinomas and squamous cell carcinomas due to the difference in the distribution of the HPV types). The susceptible compartments were further subdivided into naive and non-naive to HPV infection. DNA negative non-naive women (i.e. women who have experienced but cleared their infection) were assumed to occupy the susceptible non-naïve or naturally immune compartments. A specific precursor lesion state could regress to a less severe state, to the immune state, or to susceptible (non-naive) state, either as a result of natural regression (at rates independent of age) or of age-dependent screening and treatment (Table S5). Men were assumed only to occupy type-specific model compartments representing HPV susceptible, infected and immune. A proportion of women and men who experienced infection were assumed to develop natural immunity and be immune from further infections (we varied the proportion between 25% and 100%), however this immunity could wane with time and the individuals could then become susceptible (non-naive). The women and men who did not develop natural immunity were assumed to become fully susceptible (non-naive) to infection.

The model did not divide cancer into the four International Federation of Gynecologists & Obstetricians (FIGO) stages [[](#_ENREF_1)1], which stratify cancers cases into severity categories. However the relevant parameters were modified assuming the distribution for FIGO stages predicted by Bjorge *et al.* (Stage I: 56%, Stage II: 29% , Stage III: 12%, and Stage IV: 3% ) [[](#_ENREF_2)2]. The estimate of the excess mortality of women with cervical cancer were taken from .

**Sexual behaviour**

Age and risk group dependent partner change rates were estimated from the mean number of new partners reported by respondents to the National Survey of Sexual Attitudes and Lifestyles II [[](#_ENREF_4)4]. The low risk group represents people in the lowest 80th percentile in terms of the number of new sexual partners they acquire per year. The moderate group represents the next 15th percentile and high risk the highest 5th percentile. It was assumed that children below the ages of 12 have no sexual partners and that men had the same partner change rate as women. Data from industrialised nations suggest that mixing across risk groups is close to random with degree of assortative mixing between 0.92 and 0.65 [[](#_ENREF_5)5[,](#_ENREF_6)6]. The value was assumed to be 0.8. The degree of assortative mixing across different age groups was estimated using the difference between age of respondent and age of most recent sexual partner reported in NATSAL II and was assumed to be 0.5 [[](#_ENREF_4)4]. The mixing matrix was based on equations described in *.*

**Table S2: Number of New Partnerships per Year Stratified Age and Risk Group** [**[**](#_ENREF_4)**4].**

| **Age group Number of new partnerships per year** | | | |
| --- | --- | --- | --- |
|  | **Low** | **Intermediate** | **High** |
| 12-13 | 0.008 | 0.054 | 0.151 |
| 14-15 | 0.034 | 0.215 | 0.600 |
| 16-17 | 0.295 | 1.884 | 5.261 |
| 18-19 | 0.295 | 1.884 | 5.261 |
| 20-24 | 0.184 | 1.561 | 5.582 |
| 25-29 | 0.042 | 1.227 | 3.914 |
| 30-34 | 0.001 | 0.597 | 2.316 |
| 35-44 | 0.001 | 0.382 | 2.143 |
| 45-54 | 0.001 | 0.191 | 1.071 |
| 55-74 | 0.001 | 0.083 | 0.465 |
| The rates shown were for women, for simplity men were assumed to have the same rates.  Data taken from [^4^](#_ENREF_3) | | | |

**Table S3: Population Size and Death Rates Stratified Age Groups**.

| **Age group** | **Number of women** | **Mean death rate per 1000** | |
| --- | --- | --- | --- |
|  |  | Males Females | |
| 12-13 | 712,700 | 0.105 | 0.095 |
| 14-15 | 736,600 | 0.18 | 0.12 |
| 16-17 | 855140 | 0.405 | 0.17 |
| 18-19 | 789500 | 0.56 | 0.265 |
| 20-24 | 2,065,700 | 0.654 | 0.252 |
| 25-29 | 2,010,600 | 0.76 | 0.33 |
| 30-34 | 1,910,500 | 0.99 | 0.51 |
| 35-44 | 4,616,200 | 1.585 | 0.925 |
| 45-54 | 4,136,600 | 3.505 | 2.32 |
| 55-74 | 6,415,900 | 15.398 | 9.973 |
| Data on the population structure was taken from Office of Natural Statistics [[](#_ENREF_8)8]. Men were assumed to have the same population structure as women. | | | |

**Screening and treatment description**

Cervical screening in the UK is currently carried out using the Pap smear and we used the coverage data from the Cervical Screening Programme for England in 2005-2006 to estimate the age-specific proportion of women attending screening [[](#_ENREF_9)9]. Treatment rates were estimated based on the method described in Choi *et al*. [[1](#_ENREF_10)0]. As in Choi *et al*. [[1](#_ENREF_10)0], it was assumed the precancerous lesions for adenocarcenomas (CGIN I-III) could not be detected by screening. Treatment was assumed to always be successful and to move women to the immune or susceptible non-naive compartment. Cancer was assumed to be treated as soon as it is detected. The success rate of screening at detecting CIN I-III was estimated using median values from a meta-analysis of the sensitivity of Pap testing .

***Table S4: Screening and Treatment Rates Stratified Age Group and Neoplastic Status.***

| **Age class** | **Screening rate** | **CIN I** | **CIN II** | **CIN III** |
| --- | --- | --- | --- | --- |
| 20-24 | 11.95% | 1.85% | 6.30% | 5.12% |
| 25-29 | 23.41% | 3.62% | 12.33% | 10.02% |
| 30-34 | 25.63% | 3.96% | 13.50% | 10.97% |
| 35-44 | 26.29% | 4.06% | 13.85% | 11.25% |
| 45-54 | 23.73% | 3.67% | 12.50% | 10.16% |
| 55-74 | 11.64% | 1.80% | 6.13% | 4.98% |
| Abbreviations: CIN, cervical intraepithelial neoplasias; CGIN, cervical glandular intraepithelial neoplasia. Treatment rates represent the annual probability that a woman with a particular neoplastic state attends screening, is diagnosed as needing treatment, and is successfully treated. These parameters were estimated by a method described in Choi *et al*. [[10](#_ENREF_10)]. | | | | |

***Table S5: Lesion Specific Progression and Regression Parameters.***

|  | **HPV-16** | **HPV-18** | **Other High risk types** |
| --- | --- | --- | --- |
| **Progression (% per year)** |  |  |  |
| Normal to CIN I (% per year) | 10.5 [[12](#_ENREF_12)] | 6.8 [[12](#_ENREF_12)] | 4 [[13](#_ENREF_13),[14](#_ENREF_14)] |
| CIN I to CIN II (% per year) | 13.6 [[7](#_ENREF_7)] | 13.6 [[7](#_ENREF_7)] | 10.6* [[15](#_ENREF_15)] |
| CIN II to CIN III (% per year) | 14 [[14](#_ENREF_14),[16](#_ENREF_16)] | 14 [[14](#_ENREF_14),[16](#_ENREF_16)] | 7.9* [[15](#_ENREF_15)] |
|  |  |  |  |
| **Regression (% per year)** |  |  |  |
| HPV infection clearance (rate) | 0.57 [[17](#_ENREF_17)] | 0.57 [[17](#_ENREF_17)] | 0.57 [[17](#_ENREF_17)] |
| CIN I to normal (% per year) | 13.2 [[15](#_ENREF_15)] | 13.2 [[15](#_ENREF_15)] | 13.2 [[15](#_ENREF_15)] |
| CIN II to normal (% per year) | 17.1 [[15](#_ENREF_15)] | 17.1 [[15](#_ENREF_15)] | 17.1 [[15](#_ENREF_15)] |
| Abbreviations: CIN, cervical intraepithelial neoplasias; CGIN, cervical glandular intraepithelial neoplasia; *, represents a weighted average. The rates of progression to different lesion states were HPV type specific and both independent of age and time already spent in the state. Progression rates to adenocarcinomas and squamous cell carcinomas were HPV type specific and age dependent. Invasive cancers were assumed to be only removed with treatment. The parameters for CGIN were assumed to be the same as CIN (excluding CGIN III-to cancer progression rate). | | | |

**Quality Adjusted Life Years (QALYs)**

It was assumed that if a person’s health condition was not detected, their quality of life would be the same as a person without the condition. CIN I-III /Cancerous lesions could be detected by screening however we assumed that precancerous lesions for adenocareneas (CGIN I-III) could not be detected. Individuals who have been treated for cancer were assumed to have a reduced quality of life for 10 years after treatment.


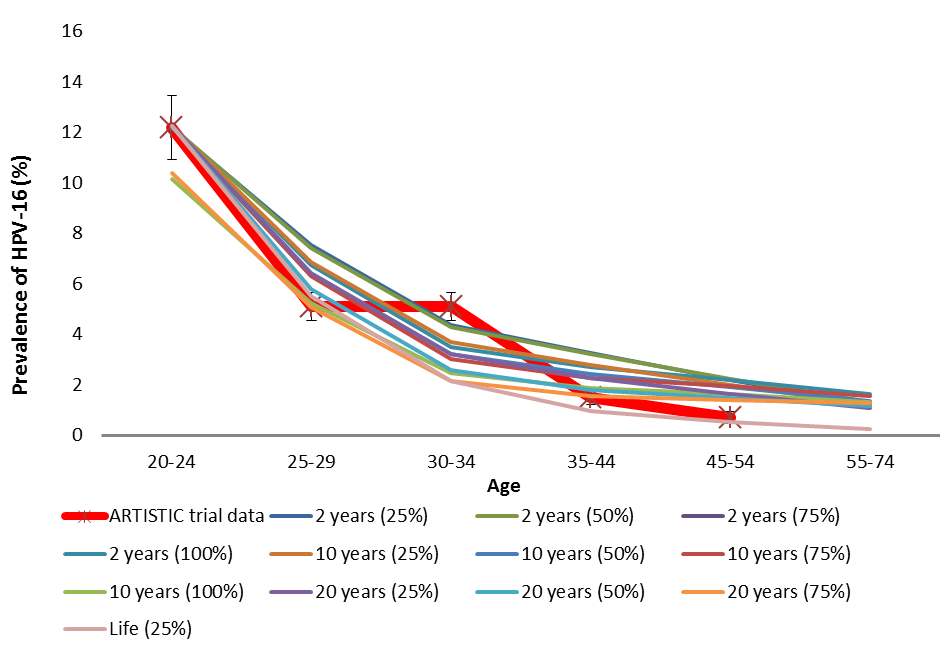

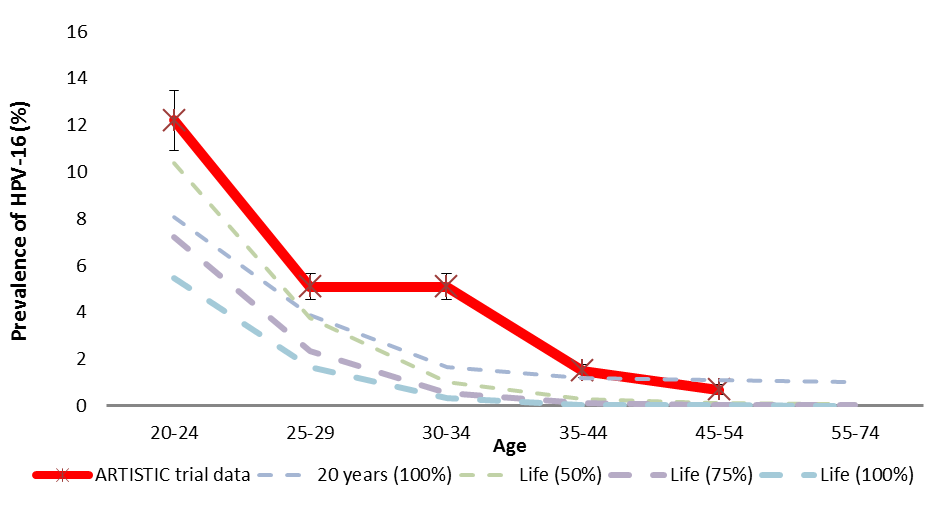


A

B

**Figure S3: Model Estimates of Age Specific Prevalence of HPV-16 Compared to the Trial Data to which it was Fitted.** *The results are shown for different assumptions of natural immunity, both the duration (2 years,10 years,20 years, life) and proportion that experience* *it (shown in brackets).**The scenarios that were included are shown in (A) and excluded are shown in (B). ARTISTIC trial data was from Kitchener et al.* [*[1*](#_ENREF_18)*8].*

**Table S6: Vaccination Coverage for Each Dose Stratified by Age.**

| **Age group** | **Vaccination Coverage** | | |
| --- | --- | --- | --- |
|  | **1st Dose** | **2nd Dose** | **3rd Dose** |
| **School Based** |  |  |  |
| 12-13 | 84.30% | 82.30% | 76.40% |
| 14-15 | 77.50% | 75.20% | 68.50% |
| **GP Based** |  |  |  |
| 16-17 | 58.10% | 53.10% | 41.70% |
| 18-19 | 55.60% | 50.30% | 38.90% |
| 20-24 | 55.60% | 50.30% | 38.90% |
| 25-29 | 55.60% | 50.30% | 38.90% |
| 30-34 | 55.60% | 50.30% | 38.90% |

Women over the age of 16 were assumed to receive their vaccine at a GP clinic. There is no data on women older than 18, so coverage is assumed to be the same as 18 year old women. All three doses of the vaccine

were assumed to administered in the same year. Data taken from a Department of Health Report [9].

**Vaccine Cost**

The cost of the vaccine was negotiated between GSK and the government and has not been released so it was varied between £20 and £40, this does not include the cost of administration. The cost of administering each dose of the vaccine in a school based program was estimated at £5.30 per dose (2011 prices), based on the estimated administration cost of hepatitis B vaccination [[2](#_ENREF_20)0]. Women over the age of 16 were assumed to receive their vaccination through general practice clinics and incur a cost of £11.87 per dose (2011 prices) for a nurse consultation [[2](#_ENREF_21)1]. When calculating the cost of vaccination, all women were multiplied by the age and dose specific coverage estimates (not just the susceptible population).

***Table S7: Effect of Altering Discount Rate on Median Incremental Cost Effectiveness Ratio.***

| **Catch-up**  **programme** | **0% Discount Rate** | **3.5% Discount Rate** | **6% Discount Rate** |
| --- | --- | --- | --- |
| 12-17 (Current) | £2,427 (2,198-4,572) | £9,476 (8,145-13,357) | £16,740 (15,311-21,047) |
| 12-19 | £8,389 (6,966-154,77) | £22,268 (17,152-33,507) | £35,618 (32,372-51,461) |
| 12-24 | £13,769 (10,944-24,962) | £36,578 (31,321-60,042) | £51,145 (47,537-79,112) |
| 12-29 | £31,819 (25,539-52,958) | £90,320 (73,986-128,491) | £114,505 (94,394-171,418) |
| 12-34 | £60,649 (48,368-109,888) | £162,040 (124,938-224,179) | £242,890 (176,640-451,085) |

Results shown assume a vaccine cost of £40 per dose (not including the cost of administration) and the vaccine provides 20 years protection. The median results are presented averaged across the estimates for different assumptions of natural immunity (The 1^st^ and 3^rd^ quartile are shown in brackets).

**Parameters estimation**

To fit the model to different immunity scenarios we used Berkeley Madonna “curve fitter” option, which uses nonlinear least-squares regression [22]. For each different immunity assumption we had a different transmission probability and a different cancer progression rate to ensure that the incidence of cancer matched the observed data in all of the scenarios we investigated. See Table S8 for the range of estimates obtained.

**Table S8: The Range of Values of HPV Type Specific Incidence and Transmission Probability.**

| **HPV 16** | Minimum | Maximum |
| --- | --- | --- |
| Transmission probability | 0.0151 | 0.159 |
| Incidence of CIN III to squamous cell carcinomas | 0.00067 | 0.31 |
| Incidence of CGIN III to adenocarcinomas | 0.00051 | 0.07 |
|  |  |  |
| **HPV 18** |  |  |
| Transmission probability | 0.0057 | 0.0194 |
| Incidence of CIN III to squamous cell carcinomas | 0.0037 | 0.15 |
| Incidence of CGIN III to adenocarcinomas | 0.0014 | 0.12 |
|  |  |  |
| **Other high risk type** |  |  |
| Transmission Probability | 0.0049 | 0.01 |
| Incidence of CIN III to squamous cell carcinomas | 0.002 | 0.085 |
| Incidence of CGIN III to adenocarcinomas | 0.0003 | 0.013 |
| Abbreviations: CIN, cervical intraepithelial neoplasias; CGIN, cervical glandular intraepithelial neoplasia. | | |


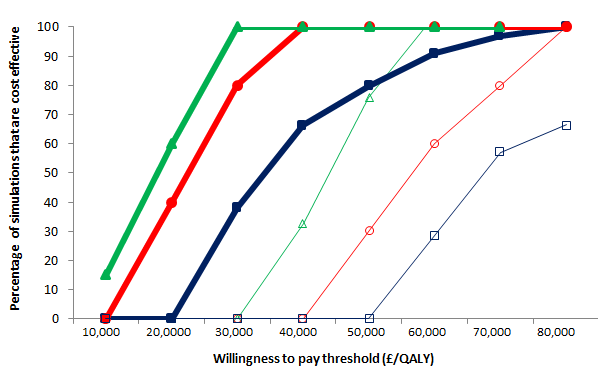


**Figure S4: Cost effectiveness acceptability curves for extending the vaccination catch-up programme up to 24 year olds (assuming a lower coverage of the school based programme targeting 12-13 year olds).** *The coverage achieved in the school based programme was assumed to be equivalent to the lower coverage achieved in the catch-up programme of 16-18 year olds (See Table S6).* *Different durations of vaccine induced immunity; Life (∆), 20 years (○), 10 years (□). Thick lines represent presence of protection to HPV non-naive women and thin the absence. The results presented assumed the vaccine cost is £20 per dose (not including the cost of administering the* vaccine) *a 100 year time horizon and 3.5% discount rate for costs and benefits. QALY: Quality adjusted life year.*

**
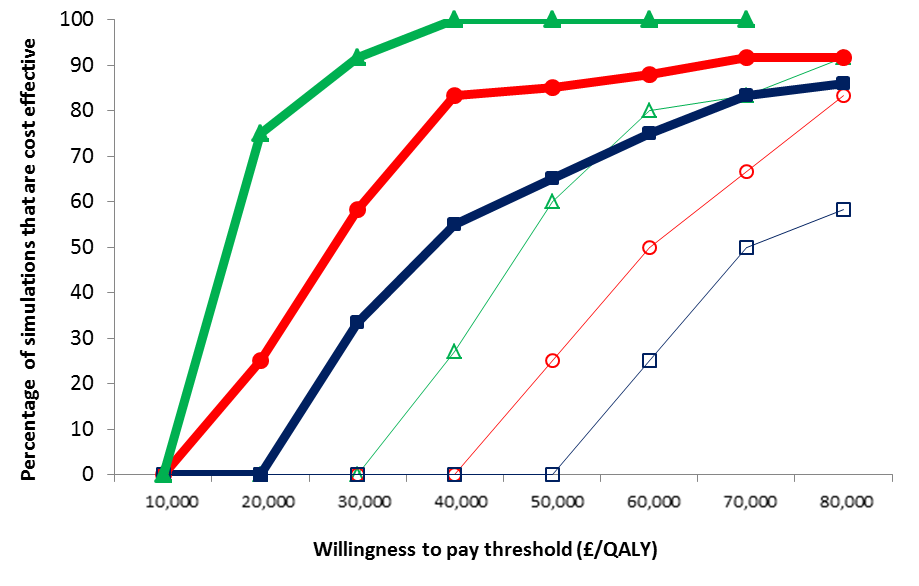
**

**Figure S5: Cost Effectiveness Acceptability Curves for Extending the Vaccination Catch-up Programme up to 24 Year Olds (Assuming 75% Vaccine Efficacy Against Infection for Non-Naive Women)**. *Different durations of vaccine induced immunity; Life (∆), 20 years (○), 10 years (□). Thick lines represent presence of protection to HPV non-naive women and thin the absence. The results presented assumed the vaccine cost is £20 per dose (not including the cost of administering the* vaccine) *a 100 year time horizon and 3.5% discount rate for costs and benefits. QALY: Quality adjusted life year.*

**
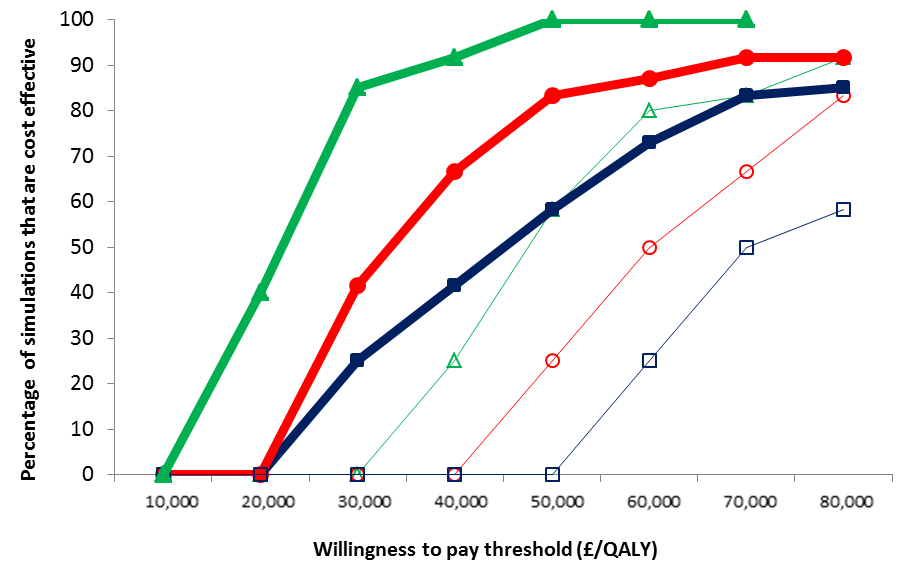
**

**Figure S6: Cost Effectiveness Acceptability Curves for Extending the Vaccination Catch-up Programme up to 24 Year Olds (Assuming 50% Vaccine Efficacy Against Infection for Non-Naive Women)**.*Different durations of vaccine induced immunity; Life (∆), 20 years (○), 10 years (□). Thick lines represent presence of protection to HPV non-naive women and thin the absence. The results presented assumed the vaccine cost is £20 per dose (not including the cost of administering the* vaccine) *a 100 year time horizon and 3.5% discount rate for costs and benefits. QALY: Quality adjusted life year.*

**References**

1. Pecorelli S, Benedet JL, Creasman WT, Shepherd JH (1999) FIGO staging of gynecologic cancer. 1994-1997 FIGO Committee on Gynecologic Oncology. International Federation of Gynecology and Obstetrics. Int J Gynaecol Obstet 65: 243-249.

2. Bjorge T, Thoresen SO, Skare GB (1993) Incidence, survival and mortality in cervical cancer in Norway, 1956-1990. Eur J Cancer 29A: 2291-2297.

3. Kohli M, Ferko N, Martin A, Franco EL, Jenkins D, et al. (2006) Estimating the long-term impact of a prophylactic human papillomavirus 16//18 vaccine on the burden of cervical cancer in the UK. Br J Cancer 96: 143-150.

4. Wellings K, Nanchahal K, Macdowall W, McManus S, Erens B, et al. (2001) Sexual behaviour in Britain: early heterosexual experience. Lancet 358: 1843-1850.

5. Granath F, Giesecke J, Scalia-Tomba G, Ramstedt K, Forssman L (1991) Estimation of a preference matrix for women's choice of male sexual partner according to rate of partner change, using partner notification data. Math Biosci 107: 341-348.

6. Laumann EO, Gagnon JH, Michael RT, Micheals S (1994) The Social Organization of Sexuality. Chicago: University of Chicago Press.

7. Elbasha EH, Dasbach EJ, Insinga RP (2007) Model for assessing human papillomavirus vaccination strategies. Emerg Infect Dis 13: 28-41.

8. Office for National Statistics (2008) Population Estimates for UK, England and Wales, Scotland and Northern Ireland, mid 2008.

9. Department of Health Statistical Bulletin (2006) Cervical screening programme, England: 2005–06. The Information Centre.

10. Choi YH, Jit M, Gay N, Cox A, Garnett GP, et al. (2010) Transmission dynamic modelling of the impact of human papillomavirus vaccination in the United Kingdom. Vaccine 28: 4091-4102.

11. Nanda K, McCrory DC, Myers ER, Bastian LA, Hasselblad V, et al. (2000) Accuracy of the Papanicolaou test in screening for and follow-up of cervical cytologic abnormalities: a systematic review. Ann Intern Med 132: 810-819.

12. Insinga RP, Dasbach EJ, Elbasha EH, Liaw KL, Barr E (2007) Progression and regression of incident cervical HPV 6, 11, 16 and 18 infections in young women. Infect Agent Cancer 2: 15.

13. Goldie SJ, Grima D, Kohli M, Wright TC, Weinstein M, et al. (2003) A comprehensive natural history model of HPV infection and cervical cancer to estimate the clinical impact of a prophylactic HPV-16/18 vaccine. Int J Cancer 106: 896-904.

14. Kataja V, Syrjanen K, Mantyjarvi R, Vayrynen M, Syrjanen S, et al. (1989) Prospective follow-up of cervical HPV infections: life table analysis of histopathological, cytological and colposcopic data. Eur J Epidemiol 5: 1-7.

15. Canfell K, Barnabas R, Patnick J, Beral V (2004) The predicted effect of changes in cervical screening practice in the UK: results from a modelling study. Br J Cancer 91: 530-536.

16. De Aloysio D, Miliffi L, Iannicelli T, Penacchioni P, Bottiglioni F (1994) Intramuscular interferon-beta treatment of cervical intraepithelial neoplasia II associated with human papillomavirus infection. Acta Obstet Gynecol Scand 73: 420-424.

17. Moscicki AB, Shiboski S, Broering J, Powell K, Clayton L, et al. (1998) The natural history of human papillomavirus infection as measured by repeated DNA testing in adolescent and young women. J Pediatr. United States. pp. 277-284.

18. Kitchener HC, Almonte M, Wheeler P, Desai M, Gilham C, et al. (2006) HPV testing in routine cervical screening: cross sectional data from the ARTISTIC trial. Br J Cancer 95: 56-61.

19. Department of Health (2011) Annual HPV vaccine coverage in England in 2009/2010 (15439). Available from: http://www.dh.gov.uk/en/Publicationsandstatistics/Publications/PublicationsPolicyAndGuidance/DH_123795 (accessed 2^nd^ February 2013

20. Wallace LA, Young D, Brown A, Cameron JC, Ahmed S, et al. (2005) Costs of running a universal adolescent hepatitis B vaccination programme. Vaccine 23: 5624-5631.

21. Curtis L (2007) Unit costs of health and social care 2007. University of Kent at Canterbury: Personal Social Services Research Unit.

22. Macey RI. and Oster GF**.**  Berkeley Madonna, version 8.0. University of California at Berkeley, Berkeley, CA. 2001.
